# Spatiotemporal and weather effects on the reproductive success of piping plovers on Prince Edward Island, Canada

**DOI:** 10.1002/ece3.11581

**Published:** 2024-08-07

**Authors:** Ryan Guild, Xiuquan Wang, Sarah Hirtle, Shannon Mader

**Affiliations:** ^1^ Canadian Centre for Climate Change and Adaptation University of Prince Edward Island St. Peters Bay Prince Edward Island Canada; ^2^ School of Climate Change and Adaptation University of Prince Edward Island Charlottetown Prince Edward Island Canada; ^3^ Island Nature Trust Charlottetown Prince Edward Island Canada

**Keywords:** Atlantic Canada, breeding population, climate change, piping plover, Prince Edward Island, spatiotemporal model, weather effects

## Abstract

Piping plovers (*Charadrius melodus* sp.) rank among North America's most endangered shorebird species, facing compounding environmental challenges that reduce habitat availability and suppress recruitment and survival rates. Despite these challenges, research on the direct effects of climate variability and extremes on their breeding ecology remains limited. Here, we employ a spatiotemporal modelling approach to investigate how location, nest timing and weather conditions influence reproductive success rates in a small breeding population of *C. m. melodus* in Prince Edward Island (PEI), Canada from 2011 to 2023. Analysis of 40 years of monitoring records from a subset of nesting sites revealed that flooding and predation have been persistent sources of reproductive failures in this population, with unexplained losses increasing in recent years. Contrary to our hypotheses, our modelled results did not support a negative impact of extreme high temperatures and strong precipitation events on reproductive outcomes. Instead, we identified a positive effect of *T*_MAX_ and no effect of strong precipitation, perhaps due to limited exposure to extreme high temperatures (>32°C) and context‐specific risks associated with precipitation‐induced flooding. However, trends in regional climate change are likely to increase exposure to—and the influence of—such factors in the near future. Our models also identified spatiotemporal variability in apparent hatch success over the study period, as well as worse hatch outcomes across popular beachgoing regions and for delayed nesting attempts. While our results offer preliminary insights into factors affecting breeding success in this population, further research will be imperative to enhance understanding of constraints on recruitment. To this end, we encourage the collection and analysis of additional time‐series data of prey populations, human activities, fine‐scale weather data and predator/flood risks associated with each nest on PEI.

## Graphical abstract

We examine the effect of weather conditions, location and nest timing on nest and brood success for an Atlantic Canada breeding population of piping plovers, an endangered migratory shorebird species. Our models identified spatiotemporal and timing effects on nest success, as well as important temperature effects during critical periods on both nest and brood success. We present additional data summaries of reported causes of nest failures within our study area, discuss potential causes of relationships identified, and highlight additional nest and site measures that are necessary to enhance modelling efforts and understanding of effects.

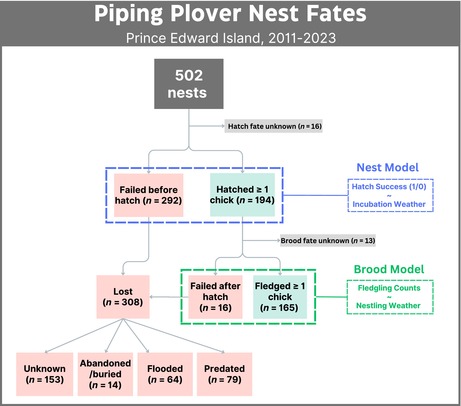

## 1 INTRODUCTION

Migratory shorebirds that cross hemispheric distances and utilize various climates and biomes during their annual cycles serve as vital indicators of global environmental change (Fiedler, 2009; Piersma & Lindström, 2004; Zöckler, 2005). Changes in their distribution, migration and reproductivity closely align with seasonal climate patterns and the availability of habitats and resources (Stutzman & Fontaine, 2015). Global declines in shorebird populations have sparked concern about their vulnerability to climate change (Koleček et al., 2021) with risk factors including habitat loss from sea level rise (SLR) and coastal erosion, hazardous migratory conditions and phenological mismatches with food sources (Galbraith et al., 2014). Recent studies of shorebird responses to the direct effects of climate change—for example, changes to temperature and precipitation—have focussed on advancements in the timing of migration and breeding that facilitate phenological mismatch, particularly for Arctic‐breeding populations that are subject to disproportionate warming (e.g. Grabowski et al., 2013; Kwon et al., 2018, 2019; Martin et al., 2018; Saalfeld et al., 2019, 2021; Saalfeld & Lanctot, 2017; Shaftel et al., 2021). Less is known about how changes to average and extreme climate conditions directly affects the reproductive success and demography of shorebirds (but see Clements et al., 2022; Cook et al., 2021; Meltofte et al., 2021; van de Pol et al., 2010; Weiser et al., 2018).

As one of the most endangered shorebird species in North America (Galbraith et al., 2014), piping plovers (*Charadrius melodus* sp.; ‘plovers’) have undergone major population declines over the past century initially attributed to early hunting pressures (Bent, 1929) and later to predation, storms, flooding, sensitivity to human disturbances and the loss of nesting habitat (Gratto‐Trevor & Abbott, 2011). An abundance of research has highlighted the vulnerability of plovers to future habitat loss from sea level rise and extreme storms (Cameron, 2022; Convertino et al., 2012; Galbraith et al., 2014; Seavey et al., 2011; von Holle et al., 2019; Zeigler et al., 2022), but far less is known about their sensitivity to the direct effects of climate change. Such effects are likely to act on key demographic parameters including survivorship, dispersal and recruitment and understanding factors that influence the latter are a primary conservation priority for the species according to Environment and Climate Change Canada (ECCC, 2021).

Predation and flooding are widely cited as major drivers of reproductive failures across plover breeding populations (e.g. Brudney et al., 2013; ECCC, 2021; Loegering & Fraser, 1995; Richardson, 1999). Additionally, a handful of studies have provided valuable insights into how breeding season environments influence reproductive success and recruitment across plover populations. For instance, the influence of habitat features like vegetation cover and substrate type on nest outcomes varies across populations, likely reflecting differences in predator communities and their search behaviours (Anteau et al., 2012; Darrah et al., 2018; Flemming et al., 1988; Patterson et al., 1991). Post‐hatch precipitation amount has also been shown to negatively affect chick survival to fledging (i.e. to 25 days post‐hatch) (Brudney et al., 2013; Gratto‐Trevor & Abbott, 2011; Harris et al., 2005; Stantial et al., 2021a) likely by inducing hypothermia and/or reducing foraging opportunities (ECCC, 2021; Stantial et al., 2021a). Conversely, droughts and temperature extremes can reduce abundances of key plover food sources (e.g. interstitial polychaetes and amphipods (Levinton, 2001; Lynn et al., 2023; Schulz & Leberg, 2019)) which can lower the fitness and survivability of both adults and hatchlings. Finally, extreme heat can also disrupt nest attendance by adults (Andes et al., 2020) that can lead to hyperthermia in exposed eggs (Amat et al., 2017) and alter foraging behaviours by chicks (Stantial et al., 2021a) that can negatively impact pre‐fledge growth rates. These effects are likely to pose significant challenges for the species as the number of extreme heat days and strong precipitation events are projected to increase across their breeding range (Seneviratne et al., 2021).

**FIGURE 1:**
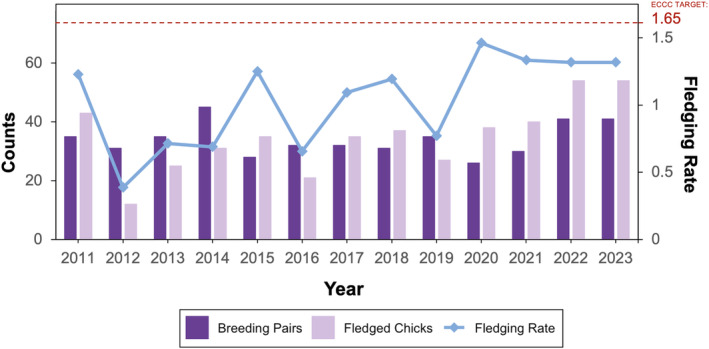
Trend in abundance, recruitment and fledging rate of piping plovers on PEI from 2011 to 2023.

Since its listing as Endangered in 1985, the breeding population of the *melodus* subspecies in Atlantic Canada has declined from 240 to 176 breeding pairs between 1986 and 2016, well below the 400‐pair threshold considered necessary for long‐term viability (ECCC, 2021). Adverse overwintering and migratory factors are suspected to have driven this decline, but considerable uncertainty remains around the factors influencing reproductive success and survivorship (ECCC, 2021). Prince Edward Island (PEI), believed to have an abundance of geomorphologically suitable nesting habitat (Amirault‐Langlais et al., 2014), has supported between 16% and 28% of the greater breeding population over the past three decades but has yet to meet established conservation targets of breeding pairs and fledging rates (Figure 1) (ECCC, 2021). In the absence of major habitat limitations, this population unit presents an ideal case study to examine the impacts of climate conditions on reproductive success. Here we examine the effect of weather and spatiotemporal factors on nest and brood outcomes for *C. m. melodus* on PEI, where observed climate changes including regional warming and altered rainfall patterns (Nawaz et al., 2023) are projected to intensify in the near future (Maqsood et al., 2023). We hypothesized that nest and brood outcomes would be negatively impacted by temperature extremes and strong precipitation events during incubation and nestling phases. Insights were first gained by evaluating reported causes of nest loss in this population. Generalized linear mixed models (GLMMs) and spatiotemporal generalized additive models (GAMs) were then used to examine the influence of location, timing and weather conditions on nest and brood outcomes for this population between 2011 and 2023.

## 2 DATA & METHODS

### 2.1 Study area & plover data

**FIGURE 2:**
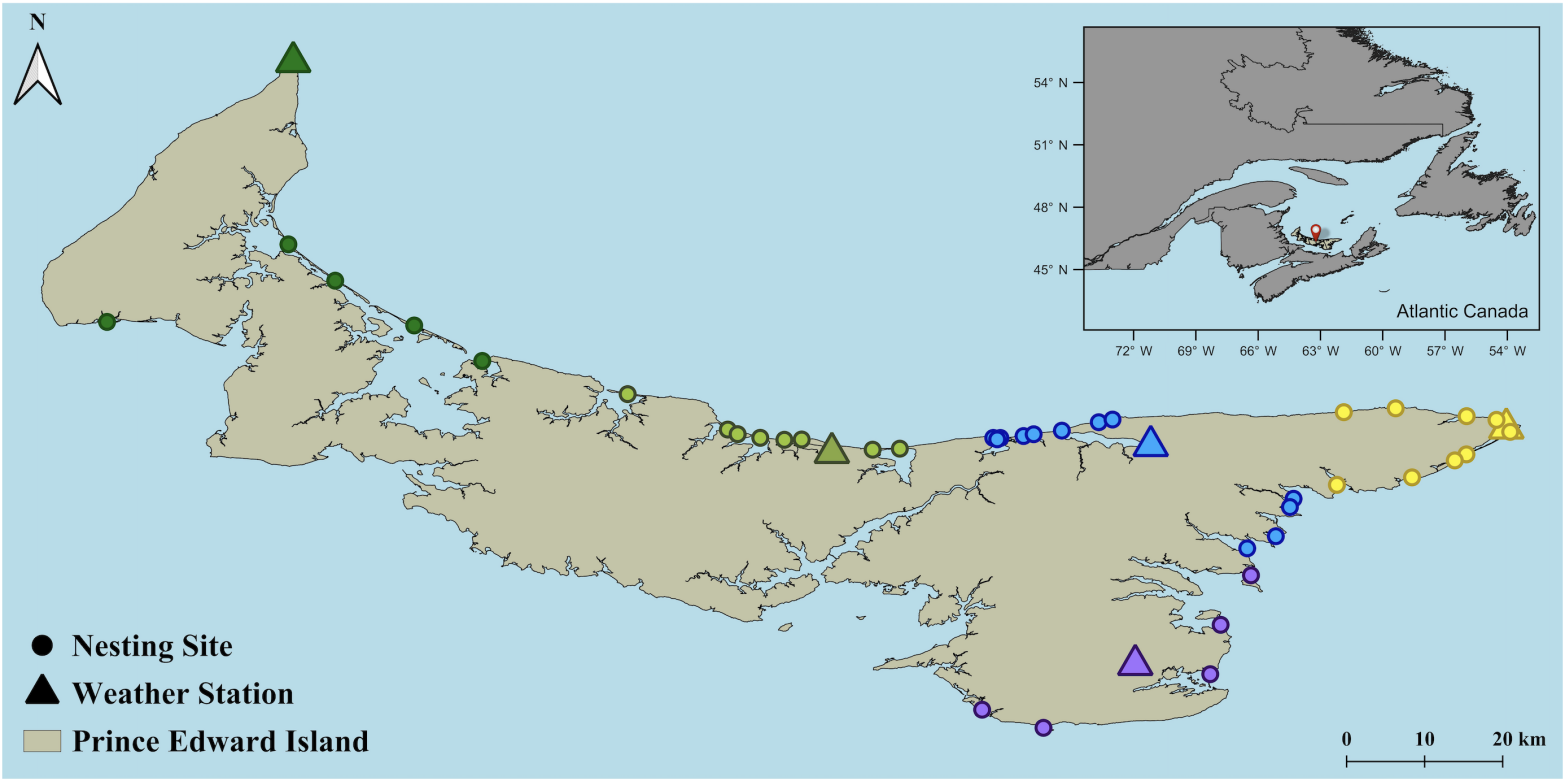
Groupings (by colour) of nesting sites to weather stations for analysis.

The population of *C. m. melodus* on PEI occupies a central position within the broader breeding range of Atlantic Canada. Each breeding season (Mar–Aug), plovers nest in supratidal habitats of mainland beaches, sandspits and barrier islands located primarily along the northern and eastern coastlines of the island (Figure 2). The vast majority of these nesting sites are separated by stretches (7.4 km average Euclidian distance; 0.7 km minimum) of unsuitable habitat, such as rocky coastlines, open water, or farmland. A small number of sites are connected by thin stretches of open sand habitat averaging 3.9 km (1 km minimum) apart. Since 1984, annual monitoring of breeding pairs, nests and broods has occurred within Prince Edward Island National Park (PEI NP), which constitutes approximately one‐fifth (*n* = 9) of nesting sites on the island. Since 2011, the conservation NGO Island Nature Trust has extended these efforts to encompass all nesting sites outside PEI NP (*n* = 33), facilitating the compilation of standardized island‐wide monitoring records thereafter. Each breeding season beginning in early March, all potential nesting sites are surveyed several times (on average 5.8 ± 0.9 visits by INT; 27.5 ± 5.5 visits by PEI NP) by teams of trained observers that search the open sand beach and dune areas for plovers and their nests and regularly cycle across nesting sites to minimize potential differences in observer effort. Upon sighting breeding activity, monitors revisit the site frequently to record nest locations, dates of loss or hatchings, and counts and fates of eggs and chicks from each nest until fledge. For the few nesting sites that host more than one breeding pair at a given time, observers utilize contextual notes on developmental stage, behaviours, and adult bands when present to keep track of (mostly unbanded) broods and render counts unambiguous. Brood losses typically occur within the first 10 days after hatching; those that disappear before fledging are attributed as lost after carefully checking nearby nesting sites (where necessary) to rule out pre‐fledge movement. Attributed losses are largely inferred from secondary evidence, such as predator tracks, beach activities, high‐tides and inclement weather conditions preceding loss.

**FIGURE 3:**
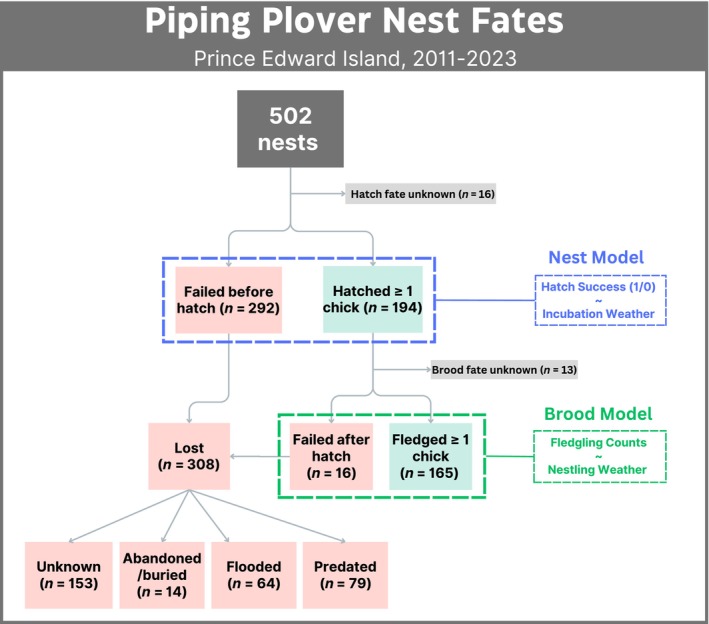
Flow chart of nest fates and associated models in this study.

We compiled a nest‐level dataset from 13 years (2011–2023) of standardized plover monitoring on PEI, yielding a total of 502 nest records (Figure 3). To examine the impact of environmental conditions and spatiotemporal factors on nest outcomes, we designed two response variables. Apparent nest success (hereafter hatch success) was employed as a binary variable (1 for at least one hatchling, 0 otherwise) to assess effects on hatchability, while brood success, defined as counts of fledglings (i.e. chicks surviving to 25 days post‐hatch) from each hatched nest, was used to test effects on juvenile recruitment. Summaries of reported and unknown reasons for nest failures from PEI NP (1984–2023) and island‐wide (2011–2023) records were additionally compiled and plotted for each observation year to visualize the attributed factors influencing reproductive success for this population.

### 2.2 Environmental data

Daily weather data during each breeding season (Mar–Aug) were obtained from five coastal weather stations across the study area based on data availability and proximity to nesting sites. Data were primarily sourced from ECCC and secondarily from Weather Underground to replace short intervals of missing values with nearby station data (within 5–12 km distance of ECCC stations). Three years of missing data from one coastal weather station (Stanhope; 2011–2013) prior to its installation were substituted with records from the nearby station of Harrington (10.5 km distance). Each nest was then paired with the closest nearby weather station, which was on average 18.9 (±2.2) km away, with a maximum distance of 60 km and a minimum of 2 km (Figure 2).

Given the sensitivity of eggs and precocial chicks to weather conditions (Beintema & Visser, 1989; Schekkerman et al., 2001), we summarized daily weather data for each nest attempt into three influential time periods according to hatch, fledge, and loss dates. These periods included (1) early‐season weather (May 1–31) coinciding with the settlement and territory‐establishment phases of breeding activity for this population, (2) incubation weather, spanning from the estimated lay date (34 days prior to hatch (Haig & Oring, 1985; Hunt et al., 2018)) to either hatch or loss date (whichever came first) and (3) nestling weather, covering the period between hatch and either fledging (25 days after hatch) or loss date (whichever came first). For each time period, the following weather variables were summarized: mean (*T*_MEAN_), maximum (*T*_MAX_) and minimum (*T*_MIN_) daily temperature, average daily temperature range (DTR) and maximum 1‐day precipitation (*P*_MAX_). These variables each conceivably influence the suitability of incubation and nestling conditions, either directly or indirectly through changes to thermoregulation, incubation or brooding behaviours, nest moisture levels or prey availability. Time‐series records of tide levels and wind speeds were not available at sufficient temporal and spatial scales to include as covariates.

### 2.3 Statistical analyses

Prior to model fitting, all continuous covariates were standardized (centred) to ensure equal weighting and to facilitate comparison of effect sizes. Collinearity among covariates was assessed using thresholds of Pearson's correlation coefficients (*r* < 0.7) and variance inflation factors (VIF < 4) for inclusion in the same model. We assessed potential non‐linear relationships between covariates and response variables by inspecting pairwise scatterplots and effective degrees of freedom estimates of smooth terms on covariates in hierarchical GAMs fitted with the R package mgcv.

#### 2.3.1 Hatch success model

For the analysis of hatch success on PEI, we selected four non‐colinear covariates for analysis that describe incubation conditions, including *T*_MAX_, *P*_MAX_, DTR and approximate lay date. *T*_MIN_ and *T*_MEAN_ were excluded from the candidate covariates due to collinearity with approximate lay date, which was retained due to superior fit in GAMs. A total sample size of 415 nests was identified after excluding those missing hatch fates (*n* = 16), approximate hatch dates (*n* = 26), spatial coordinates (*n* = 34) and incubation weather variables (*n* = 11). To test for spatial dependence, we first fit a Bernoulli random‐intercept GLMM with incubation weather covariates as fixed effects and Site and Year as random effects using the glmmTMB package in R. Spatially plotting average random intercept estimates for each site revealed evidence of small‐scale spatial dependence in hatch success, indicating that apparent hatching probability varied spatially across our study area.

To account for this spatial dependence, various spatial models of hatch success were fit as Bernoulli GAMs in R‐INLA, each with an intercept term and weather covariates as fixed effects. The spatial models excluded habitat type as it did not enhance model fit. The first model captured temporal trends in hatch success using a random walk 2 (rw2) smooth term for Year and an independent and identically distributed (iid) smooth term for Site, which assumed no spatial dependence between nests. The second model replaced the Site smoother with a Matérn correlation smoother representing the spatial random field (SRF), which captured spatial patterns in hatch success but disregarded temporal trends in the spatial effects. The third model accounted for temporal trends in the spatial effects by adding an autoregressive order 1 (AR1) term to the Matérn correlation smoother with knots at each observation year. Finally, the third model specification was refined by (1) removing the global year smoother due to negligible improvement in fit and (2) fitting potentially non‐linear weather covariates with smooth rw2 terms to address minor residual patterns in covariates. Priors, spatial range parameters and mesh size of each model are given in Appendix S1.

To evaluate model performance, we ranked each model based on DIC and WAIC estimates and calculated squared quantile residuals (SQR) by passing 1000 posterior simulations of regression parameters to the simulateResiduals function in the DHARMa package. Model fit was then assessed by plotting SQR against time, fitted values and each fixed effect and by generating spatial and temporal variograms to examine the model's ability to capture spatial and temporal dependencies in the data. For the best‐performing model, we extracted parameter mean estimates and 95% Bayes credible intervals for each fixed effect and plotted the effects of all smooth terms modelled. For each coordinate in the SRF, the spatial effects were then averaged across years to plot the average spatial pattern of hatch success over the study period. All calculations for fitting and validating models were performed in R version 4.3.2 (R Core Team, 2023) using the packages ‘sp’ (Pebesma & Bivand, 2023), ‘sf’ (Bivand et al., 2013), ‘dplyr’ (Wickham et al., 2023), ‘mgcv’ (Wood, 2017), ‘gratia’ (Simpson, 2024), ‘ggplot2’ (Wickham, 2016), ‘INLA’ (Rue et al., 2009), ‘glmmTMB’ (Brooks et al., 2017), ‘gstat’ (Gräler et al., 2016), ‘fmesher’ (Lindgren, 2023) and ‘DHARMa’ (Hartig, 2022).

The same modelling approach as above was applied using May weather covariates as fixed effects, but upon finding no covariate effects and evidence of temporal dependency between observations in the same year, we present only the results of the hatch success models with the incubation weather covariates described above.

#### 2.3.2 Fledgling count model

For the analysis of brood outcomes on PEI, which utilized counts of chicks surviving until the fledgling stage from each hatched nest, we first selected five non‐collinear nestling weather covariates for analysis, including *T*_MEAN_, *T*_MAX_, *T*_MIN_, DTR and *P*_MAX_. Approximate lay date was excluded from the candidate covariates due to collinearity with *T*_MIN_, which was retained due to superior fit (ΔAIC = 4). A total of 160 broods were identified for analysis after excluding non‐hatched nests (*n* = 294) and broods missing fledge fates (*n* = 27), nestling weather (*n* = 5) and spatial coordinates (*n* = 16).

Initial testing of per‐site random intercept estimates in Poisson GLMMs with weather covariates as fixed effects and Site and Year as random effects revealed very minor evidence for small‐scale spatial dependence in fledgling counts. To account for the potential spatial dependence, we fit random‐intercept and spatial Poisson models in INLA similar to the first two hatch success models (as described above) but could not apply a spatiotemporal model (via an AR1 term) due to a limited sample size. The spatial model did not improve fit and captured minimal evidence of spatial dependency (i.e. spatial effect multiplied fledgling counts by <5%), and both models were found to be underdispersed which cannot be accounted for in INLA. Thus, we fit a final random‐intercept Conway‐Maxwell‐Poisson GLMM that included all linear weather covariates as fixed effects and random effect terms for Site and Year. The final model was validated by calculating SQR and plotting them against time, fitted values and each covariate, and by generating variograms similar to the approach for hatch success models (as described above). Parameter estimates and standard errors (SE) were extracted from the final GLMM and effect sizes were plotted by multiplying SE by 1.96.

## 3 RESULTS

### 3.1 General descriptive results

**FIGURE 4:**
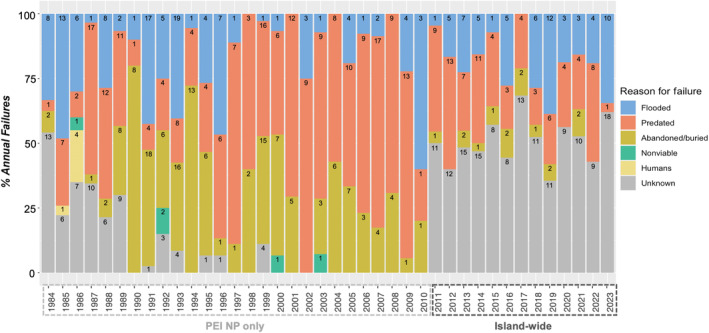
Reported reasons for reproductive failures (nests and broods) from nine PEI National Park site (1984–2010) and 41 island‐wide site (2011–2023) records. Nest counts are denoted in black at the top of each bar.

Reported reasons for reproductive failures on PEI have varied over the past four decades, but flooding and predation, and to a lesser degree burial and nest abandonment, are primarily recognized as key limiting factors (Figure 4). Since the initiation of standardized island‐wide monitoring in 2011, between 35% and 68% of annual reproductive losses occurred for unknown reasons (Figure 4). All confirmed predation events, discerned either by motion cameras or tracks within the nest cup, occurred prior to hatch when detection of such events is more feasible than when broods are mobile. Among predated nests with confirmed predators, American crows (*Corvus brachyrhynchos*) and foxes (*Vulpes vulpes*) accounted for approximately half and one‐third of predation events between 2011 and 2023 respectively. Less frequent confirmed predators included gulls (*Larus* spp.), striped skunks (*Mephitis mephitis*), raccoons (*Procyon lotor*), coyotes (*Canis latrans*), bald eagles (*Haliaeetus leucocephalus*) and common ravens (*Corvus corax*), while other suspected predators included dogs, merlin (*Falco columbarius*), American mink (*Neovison vison*) and peregrine falcons (*Falco peregrinus*). Over the study period, nests lost to either flooding (*t* = −1.8; *p* = .31), predation (*t* = −1.4; *p* = .18) or unknown factors (*t* = 1.7; *p* = .08) did not have significantly different dates of nest initiation than other lost nests. No consistent trend in approximate lay dates was identified across observation years, but peak median (7 Jun) and maximum (7 Jul) initiation dates were observed in 2023, some 9.4 and 11.7 days later (respectively) than the 2011–2023 average.

Our analysis of hatch success was based on 415 nests with known hatch/loss fates across 39 nesting sites and 13 years of observation (2011–2023) on PEI. Across nesting sites with ≥10 nests over the study period, or 73% of nest records, hatch success (% nests with ≥1 hatched chick) averaged 39% (±2.8%) with a range of 17%–67%. For the remaining sites with <10 nests, hatch success averaged 41% (±4.7%) with a range of 0%–75%. Monitored nests that were excluded from this analysis due to missing spatial coordinates (*n* = 34), approximate hatch dates (*n* = 26) and weather records (*n* = 11) showed average hatch success of 40.8% (±5.9%). Examination of incubation weather covariates mostly revealed non‐linear fluctuations over time, although *T*_MAX_ (Δmean = +0.16°C/yr; Δmax = +0.21°C/yr) and *P*_MAX_ (Δmean = +0.91 mm/yr; Δmax = −0.59 mm/yr) demonstrated the most pronounced linear trends (Table 1). Nests that experienced 90th percentile *P*_MAX_ ≥ 42.8 mm (*n* = 45) and *T*_MAX_ ≥31.3°C (*n* = 42) during incubation had average hatch success rates of 42.2% (±7.5%) and 45.2% (±7.9%) respectively.

**TABLE 1:** Characteristics of input data from 13 years of standardized nest and brood monitoring of the piping plover breeding population on PEI (2011–2023).

Year	Nests	Broods	Approx. Lay date	*T*_MEAN_		*T*_MAX_		*T*_MIN_		*P*_MAX_		DTR	
				*i*	*n*	*i*	*n*	*i*	*n*	*i*	*n*	*i*	*n*
2011	32	18	29 May	–	16.5	26.1	27.4	4.2	8.9	16.5	24.4	7.9	8.1
2012	30	5	24 May	–	16.5	26.6	27.4	3.3	6.8	18.5	40.0	9.5	9.0
2013	34	9	28 May	–	18.4	26.7	30.3	4.3	8.6	28.1	30.2	8.8	9.2
2014	33	10	31 May	–	20.0	26.9	29.4	3.1	11.4	17.5	21.2	8.5	8.3
2015	22	8	1 Jun	–	17.7	25.6	28.5	4.4	9.4	27.7	18.9	8.4	7.8
2016	24	9	31 May	–	17.9	27.0	28.0	2.5	8.6	13.8	15.5	8.6	8.0
2017	23	11	2 Jun	–	18.8	27.5	27.7	3.5	8.6	21.1	20.7	8.3	9.0
2018	36	15	29 May	–	20.8	28.4	30.0	0.9	10.6	26.4	23.8	9.6	8.7
2019	43	11	3 Jun	–	17.9	25.9	29.3	4.8	10.3	32.9	24.4	7.7	6.7
2020	29	13	28 May	–	19.3	30.0	30.6	2.2	9.4	12.3	17.2	9.3	7.8
2021	22	13	1 Jun	–	18.4	30.5	29.6	2.7	6.7	24.3	25.4	10.5	9.3
2022	40	21	25 May	–	18.9	26.7	29.0	3.9	11.8	25.4	35.3	7.9	7.2
2023	47	17	1 Jun	–	19.8	27.8	29.6	4.8	11.4	35.4	44.2	7.4	6.9
Total	415	160											

*Note*: Data summarized includes mean lay dates, number of nests and broods analyzed and averages of maximum (*T*_MAX_) and minimum (*T*_MIN_) temperatures (°C), daily temperature range (DTR) and maximum daily precipitation (*P*_MAX_, mm) during incubation (*i*) and nestling (*n*) periods. Excluded samples are described in Section 2.3.

Our analysis of fledgling counts was based on 160 broods of known fledging/loss fates across 32 nesting sites and 13 years of observation (2011–2023). Across nesting sites with ≥5 broods over the study period, or 69% of records, the average number of fledglings per hatched nest was 2.2 (±0.1), ranging from 0 to 4. For the remaining sites with <5 broods, the average number of fledglings was 2.2 (±0.2) with a range of 0 to 4. Broods with known fledge fates that were excluded from analysis due to missing spatial coordinates (*n* = 16) and nestling weather records (*n* = 5) had on average 1.9 (±0.3) fledglings per hatched nest with a range of 0 to 4. Several candidate covariates describing nestling weather conditions revealed pronounced linear trends over time, including *T*_MAX_ (Δmean = +0.14°C/year, Δmax = +0.05°C/year), *T*_MIN_ (Δmean = +0.17°C/year, Δmax = +0.19°C/year), *P*_MAX_ (Δmean = +0.92 mm/year, Δmax = +0.24 mm/yr) and *T*_MEAN_ (Δmean = +0.18°C/year, Δmax = +0.16°C/year) (Table 1). During the study period, 16 broods experienced 90th percentile *P*_MAX_ ≥ 48.4 mm (average 2.7 ± 0.3 fledglings), 25 broods encountered 90th percentile *T*_MAX_ ≥ 31.3°C (2.6 ± 0.2 fledglings) and 19 broods experienced 10th percentile *T*_MIN_ ≤ 5.4°C (2.8 ± 0.3 fledglings).

### 3.2 Hatch success models

#### 3.2.1 Model selection

The spatiotemporal GAM of hatch success showed considerably lower DIC and WAIC values than either the spatial or random‐intercept (non‐spatial) GAMs (Table 2). The spatiotemporal GAM was better fit with approximate lay date than with the collinear covariate of *T*_MIN_ or *T*_MEAN_, and inclusion of habitat type as a fixed effect did not improve model fit (Table 2). The best‐performing model was a spatiotemporal GAM with linear fixed effects of *T*_MAX_, DTR and approximate lay date and smooth terms on *P*_MAX_ and the spatial field (Table 2). This model did not contain any remaining spatial or temporal correlation in the SQR.

**TABLE 2:** Model selection results for hatch success and fledgling counts of piping plovers on PEI, 2011–2023.

Response variable	Model^a^	*K* ^b^	DIC	WAIC
Hatch success^c^	T_MAX[i]_ + DTR_[i]_ + f(P_MAX[i]_)^1^ + date + f(w_AR1_)^3^	37.7	424.8	424.9
	T_MAX[i]_ + DTR_[i]_ + P_MAX[i]_ + date + f(w_AR1_)^3^	37.1	427.3	427.7
	T_MAX[i]_ + DTR_[i]_ + P_MAX[i]_ + date + f(yr)^1^ + f(w_AR1_)^3^	38.8	425.9	426.9
	T_MAX[i]_ + DTR_[i]_ + P_MAX[i]_ + date + f(yr)^1^ + f(w)^3^	15.7	443.4	443.6
	T_MAX[i]_ + DTR_[i]_ + P_MAX[i]_ + date + f(yr)^1^ + f(site)^2^	16.4	444.8	445.2
	T_MAX[i]_ + DTR_[i]_ + P_MAX[i]_ + date + f(yr)^1^ + f(site)^2^ + Habitat	18.7	448.1	448.9
	T_MAX[i]_ + DTR_[i]_ + P_MAX[i]_ + T_MIN[i]_ + f(yr)^1^ + f(site)^2^ + Habitat	19.4	453.8	454.9
	T_MAX[i]_ + DTR_[i]_ + P_MAX[i]_ + T_MEAN[i]_ + f(yr)^1^ + f(site)^2^ + Habitat	20.5	473.9	474.9
2 Fledgling counts^d^	T_MAX[n]_ + DTR_[n]_ + P_MAX[n]_ + T_MIN[n]_ + T_MEAN[n]_ + yr^4^ + site^4^	7.0	–	–
	T_MAX[n]_ + DTR_[n]_ + P_MAX[n]_ + T_MIN[n]_ + T_MEAN[n]_ + f(yr)^1^ + f(w)^3^	6.2	542.2	537.5
	T_MAX[n]_ + DTR_[n]_ + P_MAX[n]_ + T_MIN[n]_ + T_MEAN[n]_ + f(yr)^1^ + f(site)^2^	5.8	541.8	537.5

^a^ Explanatory variables include linear standardized continuous variables of maximum daily temperature (T_MAX_), maximum daily precipitation (P_MAX_), mean daily temperature range (DTR), and minimum daily temperature (T_MIN_) during incubation ([i]) and nestling ([n]) periods, as well as smooth terms (f) on year (yr), nesting location (site), P_MAX_, and the spatial correlation term of nest coordinates with (w_AR1_) and without (w) a first‐order autoregressive structure. Superscript numbers indicate smoothing term applied, including (1) a random walk 2 (rw2) term for temporal trends, (2) an independent and identically distributed (iid) term for unstructured variability, and (3) a Matérn correlation term to capture spatial dependence.

^b^ Number of effective parameters in the model.

^c^ Models fitted as Bernoulli GAMs in INLA.

^d^ Best‐fit model (first listed) is a Conway‐Maxwell Poisson GLMM fit in glmmTMB to account for underdispersion. Superscript (4) refers to random‐intercept terms for the variables Year and Site. Second‐ and third‐listed models fitted as spatial and random‐intercept (non‐spatial) Poisson GAMs in INLA.

#### 3.2.2 Model inference

**FIGURE 5:**
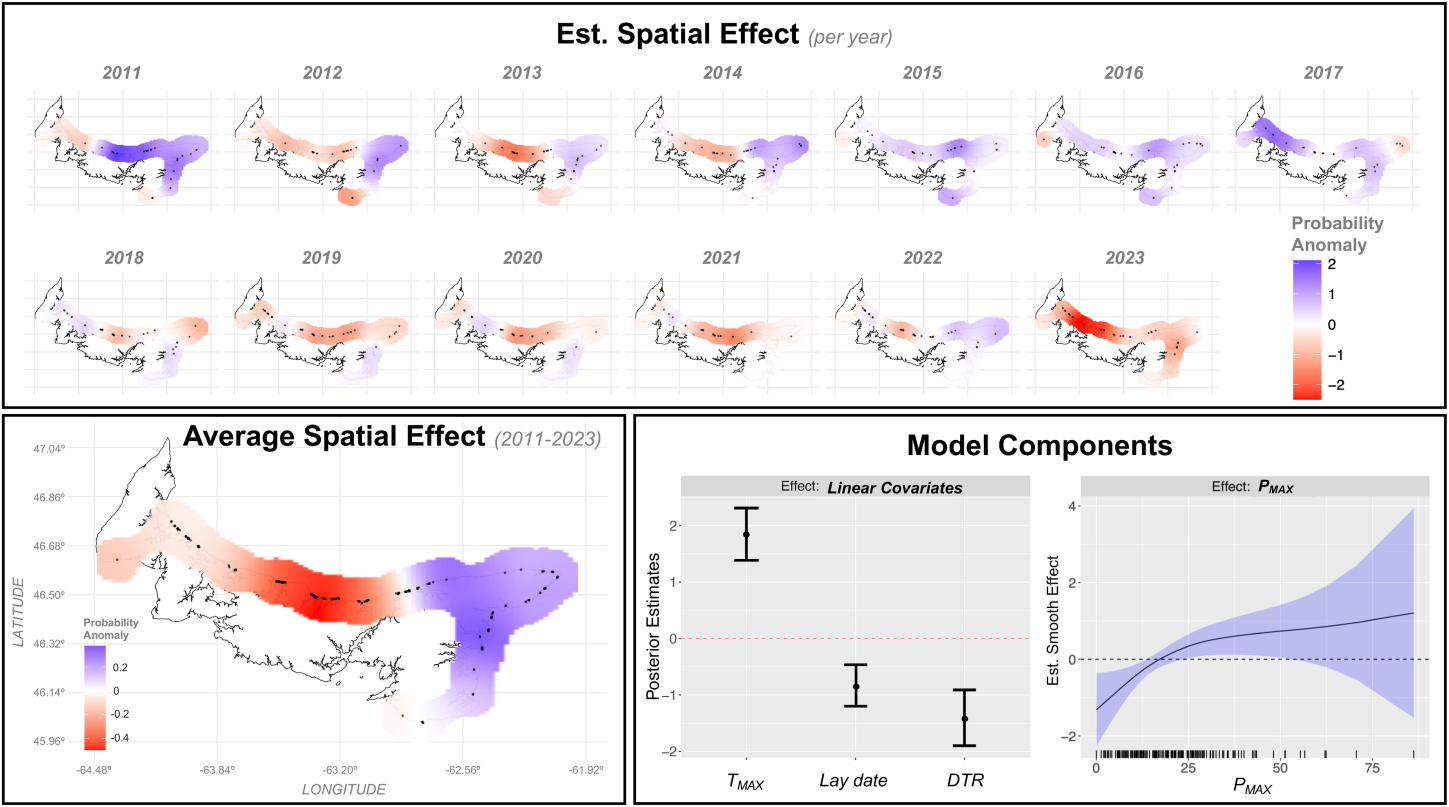
Results from the top‐ranked spatiotemporal model of hatch success on PEI, 2011–2023. Posterior mean of the SRF per year (*top*) and averaged over observation years (*bottom left*), and posterior mean estimates of fixed and smooth terms with 95% Bayes credible intervals (*bottom right*). SRF depicts nest coordinates (black) and above‐ (blue) and below‐average (red) apparent probabilities of hatch success on the log‐odds scale.

Estimated effects from the best performing model of hatch success are presented in Figure 5. Of the fixed effects, *T*_MAX_ demonstrated the strongest positive effect on hatch success (*β* = 1.85; 95% CI = 1.4 to 2.32), followed by strong negative effects of DTR (*β* = −1.4; 95% CI = −1.9 to −0.94) and approximate lay date (*β* = −0.84; 95% CI = −1.2 to −0.51) (Figure 5). The *P*_MAX_ smoother exhibited a subtle positive non‐linear effect on hatch success that may have been influenced by a small number of nest samples exposed to high values of *P*_MAX_ (Figure 5). Notably, parameter estimates and 95% CIs of the fixed effects did not differ substantially between alternative models.

The SRF revealed considerable inter‐annual variation in hatching success across both space and time (Figure 5). Moderate spatial dependency was identified up to 15 km between nest observations, implying a tendency for nearby locations to share similar log‐odds of hatch success. Blue areas indicate locations favouring conditions for hatch success (where +1 indicates apparent hatch probabilities of (1 + exp(−(+1)))^−1^, or 73%), while red areas indicate the opposite (where −1 indicates probabilities of (1 + exp(−(−1)))^−1^, or 27%). Plotting the temporal average of the spatial effects revealed hotspot areas of above‐ and below‐average log‐odds of hatch success during the study period (Figure 5). ‘Important’ or ‘strong’ spatial effects are regions with SRF values above +1.4 and below −1.4, determined by doubling the posterior standard deviation estimates (*σ* = 0.7; Figure S1). This suggests that most of the estimated spatial effects on hatch success are of moderate or weak strength (Figure 5).

### 3.3 Fledgling count models

#### 3.3.1 Model selection

The spatial GAM of fledgling counts from each hatched nest estimated marginal spatial effects (i.e. scaled probabilities by <5%), exhibited underdispersion and did not improve fit beyond the random‐intercept (non‐spatial) GAM (Table 2). Thus, a random‐intercept Conway–Maxwell–Poisson GLMM of fledgling counts was fit with nestling weather variables as linear fixed effects and Year and Site as random effect terms (Table 2). This model did not contain any spatial or temporal correlation in the SQR.

#### 3.3.2 Model inference

**FIGURE 6:**
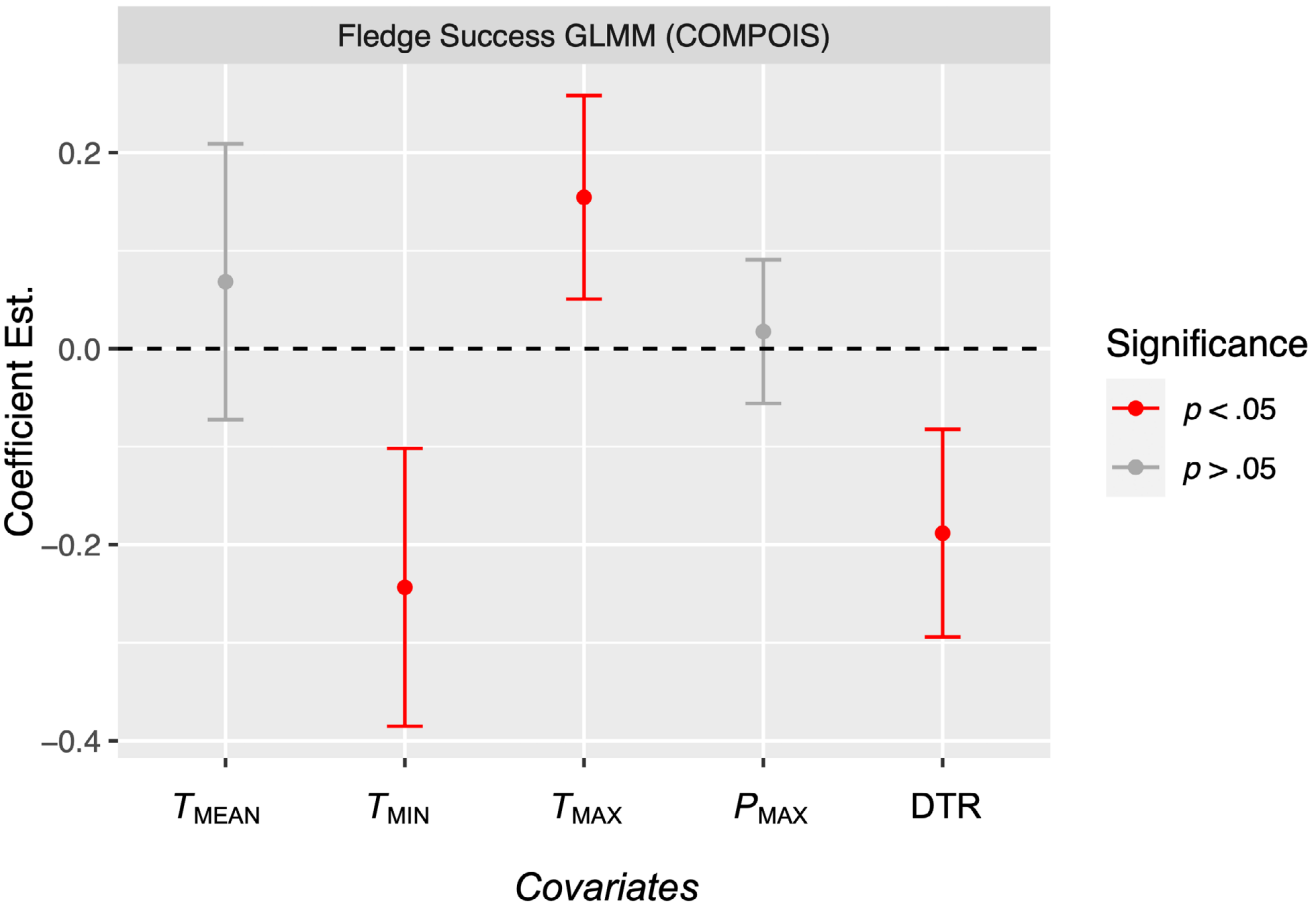
Effect sizes from GLMM of fledgling counts on PEI, 2011–2023. 95% confidence intervals plotted as standard errors * 1.96.

Among the fixed effects in the final GLMM, fledgling counts showed a significant positive relationship with *T*_MAX_ (*β* = 0.15, 95% CI = 0.051 to 0.26) and significant negative relationships with *T*_MIN_ (*β* = −0.24, 95% CI = −0.10 to −0.39) and DTR (*β* = −0.19, 95% CI = −0.083 to −0.29) (Figure 6). Unstandardized coefficient estimates on the real scale indicate that for each unit increase in *T*_MAX_, *T*_MIN_ and DTR, fledgling counts from hatched nests are estimated to change by approximately +0.32, −0.79 and −0.30 counts on average, respectively, holding all other variables constant.

## 4 DISCUSSION

This study examines various factors affecting the reproductive success of *C. m. melodus* nesting on PEI. Using partial (est. 1984) and island‐wide (est. 2011) monitoring records, we find that flooding and predation have been longstanding sources of reproductive failures for this population to varying degrees between years, consistent with previous claims for this population (e.g. Elliott‐Smith et al., 2015; Thomas & Lajeunesse, 2004) and others (e.g. Claassen et al., 2014; Cohen et al., 2010; Doherty & Heath, 2011; Engley et al., 2004; Gratto‐Trevor & Abbott, 2011; Lauro & Tanacredi, 2002; Richardson, 1999; Weithman et al., 2019). A lack of data on predator‐ and flood‐risks to nests, such as proximity to swash line or trees, nesting elevation and fine‐scale tidal records, currently prevents in‐depth analyses of these factors on PEI, but insights can be gained from previous research and modelled results from this study.

For one, the use of predator exclosures on PEI between 1988 and 2011 was associated with higher rates of adult mortality and nest abandonment as predators learned to associate exclosures with prey (Barber et al., 2010; Beaulieu et al., 2014)— an effect found in other (Murphy et al., 2003; Stantial et al., 2021b) but not all populations (Anteau et al., 2022). Similarly, a common assumption on PEI (and elsewhere (ECCC, 2021; Maslo & Lockwood, 2009)) is that predators are attracted to popular beachgoing nesting sites by human food waste. Our top‐ranked hatch success model estimated dynamic spatiotemporal variation in hatch outcomes, suggesting potential non‐uniformity in environmental influences (e.g. weather, habitat quality, threats) over space and time. The temporal average of these effects, however, reveals apparent hatching probabilities that generally align with patterns of beach use on PEI. The highly trafficked northcentral sites near dense populations on the island were associated with below‐average hatch outcomes across observation years, while the less trafficked eastern nesting sites exhibited above‐average hatch outcomes across observation years. It is possible that elevated predator densities around popular beachgoing sites lead to greater predation rates of nests (29% of losses in northcentral sites vs. 22% of losses elsewhere), as speculated in (Beaulieu et al., 2014; Elliot‐Smith et al., 2015), and/or that proximity to humans and beachgoing activities alter adult plover behaviours that lowers reproductive outcomes at these sites, as discussed in (Burger, 1991; Cairns, 1982; Flemming et al., 1988). Conversely, our findings of negligible evidence of spatial patterns in fledging counts among hatched nests suggests that spatial factors affecting chick survival may be relatively more uniform across PEI or less influential than those impacting hatching. Attributing the cause of the spatial effects on hatch success requires additional monitoring data of predator populations and beach use patterns across plover nesting sites on PEI.

On the issue of nest flooding, plover nesting habitats bordering the Gulf of St. Lawrence, where PEI is located, are characterized by relatively flatter and wider profiles compared to other regions along the Atlantic Coast (Boyne et al., 2014). While such habitats are generally favoured by plovers (Maslo et al., 2011) likely due to reduced detection risk by predators (Burger, 1987; Espie et al., 1998), nests initiated on these beaches have limited elevation buffers that render them susceptible to inundation from storm surges, high tides and heavy rainfall. Attempts to temporarily rescue flooded nests soon after submersion have proven successful within PEI NP (McNight et al., 2004; Thomas & Lajeunesse, 2004) but deploying such efforts at an island‐wide scale presents a logistical challenge and may be insufficient to curtail flood‐related losses on PEI considering growing trends of sea level rise and storm surge events in the region (Greenan et al., 2018). More targeted approaches of recording elevation buffer for each discovered nest, tracking tide levels with predictions and local gauges and annually measuring profiles of nesting habitat may serve to identify at‐risk nests and deploy rescue attempts accordingly.

The proportion of unexplained reproductive failures has increased substantially since island‐wide monitoring began in 2011, particularly for pre‐hatch losses that constitute the bulk of reproductive failures on PEI (Figure 3). This ambiguity is particularly pronounced outside of the National Park, where unknown causes accounted for an average of 64% (± 4.3%) of annual losses. However, unexplained failures within the nine National Park sites have also risen since 2011 to account for 20% (±4.8%) of annual losses on average. While it is nearly certain that the primary loss reasons discussed contribute to some of these unknowns, it is also possible that additional yet‐unaccounted‐for environmental factors influence reproductive success within this population.

We first hypothesized that extreme temperatures during incubation and nestling phases would negatively affect nest and brood outcomes for this population. This was informed by observations of high temperatures reducing nest attendance in *circumcinctus* plovers (Andes et al., 2020), and of extreme heat impairing foraging by precocial broods of *melodus* plovers (Stantial et al., 2021a) and decreasing the abundance of key plover prey populations in Atlantic Canada (Butler et al., 1996; Moore & Francis, 1986). Additionally, while lethal temperatures for *C. melodus* (sp.) eggs and broods have not been definitively identified, research on Little Terns (*Sternula albifrons*) within the same taxonomic order (Charadriiformes) suggests that unattended eggs can experience hyperthermia when ambient temperatures exceed 32°C (Amat et al., 2017).

Despite this context and contrary to our initial hypothesis, our models identified strong positive effects of maximum incubation and nestling temperatures on nest and brood outcomes between 2011 and 2023. These relationships are largely influenced by worse outcomes at cooler maximum temperatures, as evidenced by markedly below‐average outcomes at 10th percentiles of *T*_MAX_ (10% hatch, 60% fledge rates) compared to near‐average outcomes at 90th percentiles of *T*_MAX_ (45% hatch, 90% fledge rates). Less than 2% of nests (6/415) and 1% of broods (1/160) analyzed experienced *T*_MAX_ exceeding 32°C, indicating that most samples analyzed were not exposed to peak breeding season temperatures that can exceed 34°C on PEI. It is important to note that the ambient air temperature indices used are not direct measures of microscale sand temperatures that eggs and chicks are exposed to, which may underestimate their exposure to extreme heat. While these results cannot speak to the behavioural impacts of high ambient temperatures on plovers, as in (Andes et al., 2020; Stantial et al., 2021a), they indicate that, below a certain threshold, warmer windows of *T*_MAX_ were more conducive to reproductive success than cooler windows during the study period. Daily temperature range was also implicated in our models as having a strong negative effect on nest and brood outcomes. Taken together, we speculate that until suboptimal temperature extremes become commonplace for incubation and nestling phases of this population, increased prey availability at warmer temperatures (Hunt et al., 2017; Lynn et al., 2023) and lower energetic costs associated with warmer, stable temperatures (Taff & Shipley, 2023) may alleviate constraints on foraging and thermoregulation, leading to greater reproductive outcomes.

It is important, however, to interpret these results in light of ongoing climatic changes in our study area. The positive trend in average and maximum *T*_MAX_ observed in this study, which echoes trends in historical records (Nawaz et al., 2023) and future projections (Maqsood et al., 2023) on PEI, will likely expose a growing number of nests and broods to suboptimal conditions in the future, both in terms of thermoregulatory limits and prey availability which can decline rapidly at extreme high temperatures (Butler et al., 1996; Moore & Francis, 1986). This possibility is of particular concern if this population does not begin to advance their timing of breeding away from peak summer temperatures. Additional data on physiological tolerances of *C. melodus* (sp.) and tracking of nest cup temperatures will be crucial to understand the sensitivity of this and other plover populations to temperature extremes.

Our finding of a significant negative relationship between *T*_MIN_ and fledgling counts appears to conflict with the interpretation that warmer (cooler) temperatures are associated with better (worse) outcomes in this population. It is possible that the effect of *T*_MIN_ on fledgling counts is confounded by strong collinearity with lay date and that the relationship identified is more reflective of a decline in reproductive outcomes as the breeding season progresses—a strong effect that was observed in our hatch success model and that is commonly reported across plover populations (Brudney et al., 2013; Claassen et al., 2014; Harris et al., 2005; Saunders et al., 2012). It may be the case that early nesting attempts on PEI involve a higher proportion of older, more experienced breeders (as observed in (Saunders et al., 2012)) and/or benefit from lower predation pressures (as observed in (Kruse et al., 2001)), factors that could enhance outcomes of early nests but have yet to be examined for this population.

We also hypothesized that extreme precipitation events during incubation and nestling phases would be associated with worse reproductive outcomes in this population. This was based on observations that chick survival in both *circumcinctus* (Brudney et al., 2013; Harris et al., 2005; Harris & Lamont, 1991) and *melodus* (Stantial et al., 2021a) plovers is negatively impacted by post‐hatch precipitation amount, speculated to be caused by reduced foraging ability or exposure to hypothermia. Contrary to our hypothesis, *P*_MAX_ was estimated to have no discernable effect on fledgling counts. Instead, it exhibited a positive effect on hatch success, albeit weakening at high maximum daily precipitation levels (>30 mm; 28% nests) and ceasing at extreme levels (>50 mm; 7% nests). We speculate that the positive effect on hatch success at lower precipitation amounts (<30 mm) may be related to reduced prey availability during dry periods (Levinton, 2001; Schulz & Leberg, 2019) which compels adults to forage more and attend nests less, leading to worse hatch outcomes. Nests exposed to high single‐day precipitation amounts were associated with near‐average rates of hatch success (40%) but higher‐than‐average incidences of flooding (68% attributed losses at high *P*_MAX_ vs. 40% all attributed losses). Interestingly, of the four nests subject to the greatest single‐day precipitation events (>70 mm), three had 100% hatch rates without intervention and each successfully fledged at least one chick. While our weather station data may not fully capture the microscale precipitation maximums experienced by each nest or brood, the fact that 82% (37/45) and 94% (15/16) of 90th percentile *P*_MAX_ records for incubation and nestling periods (respectively) correspond with high precipitation amounts (>25 mm) at neighbouring weather stations supports the data's ability to capture local‐scale precipitation patterns. Taken together, we find that not all nests and broods exposed to strong precipitation events on PEI face similar risks of failure, and speculate that certain characteristics of nesting habitat, such as elevation, slope or substrate composition may predetermine their vulnerability to such events.

The approach and interpretations of this study are subject to several additional limitations. A potential shortcoming of our survey data is disparate survey efforts between monitoring sites, particularly between sites within—and outside of—the National Park. Limited nest monitoring metadata (i.e. lacking visitation dates and times) prevented the use of nest survival (e.g. Mayfield) models and the assessment of detection probabilities that can be influenced by temperature and thus potentially bias estimates of reproductive success. However, a detectability study on PEI across 14 monitoring sites found no mean improvement in the detectability of nests with additional survey effort (Elliot‐Smith et al., 2015). Furthermore, our exclusion of nests and broods without hatch dates or that were not monitored frequently enough to determine fates or estimate loss dates enhanced the reliability of the survey data used. The use of extrapolated dates for nest initiation (34 days prior to hatch (Haig & Oring, 1985; Hunt et al., 2018)) and fledging (25 days post‐hatch) also introduces potential inaccuracies in the assignment of weather conditions to nests and broods, particularly for maximum and minimum weather indices that are sensitive to inaccurate window dates. However, this approach was justified for several reasons. First, fledging dates on PEI are already determined by extrapolating (25 days) from hatch dates. Second, hatch dates are more reliable than discovery dates of full clutches for extrapolating laying dates, as aging eggs through floating or candling are more invasive and can carry a wide margin of error. Lastly, the majority (>80%) of maximum and minimum weather estimates correspond to dates that were not within 2 days of the outer bounds of incubation/nestling windows, minimizing the impact of minor dating inaccuracies on these variables. Our study also does not consider several key factors on the breeding grounds that are important to nest and brood outcomes, including nest‐level measures of elevation/slope, degree of camouflage, distance to shelter/trees/swash line/wet sand and nest cup materials, as well as time‐series measures of predator/prey populations, human activities, tide levels and wind speeds (Brudney et al., 2013; Catlin et al., 2019; Loegering & Fraser, 1995; Patterson et al., 1991; Schulz & Leberg, 2019). These data are either gradually becoming available (e.g. recent and ongoing installations of new weather stations) or are encouraged to be added to standardized monitoring efforts to enhance future analyses of climate‐related impacts on this population. The aforementioned scale and proxy issues associated with assigning weather station data to nest conditions may also be improved in the future with the use of new local weather stations, nest‐level cameras (for precipitation and loss attribution) and iButton or thermocouple loggers which have proven feasible in measuring nest temperatures and rates of nest attendance in plovers (Andes et al., 2020; Schneider & McWilliams, 2007).

## 5 CONCLUSION

*Melodus* or Atlantic Coast piping plovers face a multitude of environmental challenges across their migratory cycles, ranging from habitat limitations to human disturbances and climate change. With fewer than 2000 breeding pairs remaining, it is imperative to understand constraints on key demographic parameters, including recruitment rates. This study examines a dimension of these challenges for a small breeding population in Eastern Canada, where the required reproductive rates to maintain population stability are the highest of any population unit (Calvert et al., 2006; Hecht & Melvin, 2009) and have yet to be achieved (ECCC, 2021). We demonstrate that reproductive outcomes for piping plovers on PEI are mainly constrained at the nest stage, primarily due to unidentified causes and to some extent by predation, flooding and abandonment/burial. Our modelled results reveal lower hatch success rates across popular beachgoing sites and for delayed nesting attempts. However, they offer no evidence to support our hypotheses that inclement weather conditions worsen reproductive outcomes. We speculate that the strong positive effect of maximum temperatures during critical periods may only apply below thresholds of extreme heat (<32°C), but caution that trends on PEI may increasingly subject more nests and broods to such extremes in the near future. Moreover, while strong precipitation events during critical periods did not influence the modelled outcomes, nests exposed to them had higher‐than‐average flooding incidences, hinting at potential variations in risk based on specific nest site characteristics. These findings offer preliminary insights into factors limiting reproductive success in this breeding population, highlighting potential influences on predation and flood risks and speculating on the cause of observed weather effects. Further research will require additional nest‐ and site‐level measures to enhance understanding of threats to reproductive success and to develop targeted conservation efforts supporting recruitment rates in this population.

## AUTHOR CONTRIBUTIONS

**Ryan Guild:** Conceptualization (equal); data curation (equal); formal analysis (lead); funding acquisition (equal); methodology (lead); software (lead); visualization (lead); writing – original draft (lead); writing – review and editing (lead). **Xiuquan Wang:** Conceptualization (equal); formal analysis (supporting); funding acquisition (equal); investigation (supporting); methodology (supporting); resources (equal); supervision (lead); writing – original draft (supporting); writing – review and editing (supporting). **Sarah Hirtle:** Data curation (equal); methodology (supporting); resources (supporting); writing – review and editing (supporting). **Shannon Mader:** Conceptualization (equal); data curation (equal); funding acquisition (equal); methodology (supporting); resources (equal); writing – review and editing (supporting).

## Supporting information


Appendix S1.

## Data Availability

The data used by this study are available from Environment and Climate Change Canada and Island Nature Trust. Restrictions apply to the availability of these data, which were used under license for this study.
