# Supplementary material for: Spatiotemporal and weather effects on the reproductive success of piping plovers on Prince Edward Island, Canada
Source: Ecol Evol. 2024 Aug 7;14(8):e11581. doi: 10.1002/ece3.11581 (PMC11303845; doi:10.1002/ece3.11581)
Supplement: Supplementary file 1 — Appendix S1. [file ECE3-14-e11581-s001.zip › Appendix S1.docx]

**Appendix S1**

**Fig. S1.** Posterior standard deviation (SD) of the SRF at each knot (i.e., observation year) from the top-ranked spatiotemporal model of hatch success. SD are around 0.70 at nest locations (depicted in black), implying that spatial effects beyond 2*SD, or +1.4 and -1.4, indicate “important” or “strong” spatial effects.

*Hatch Success Models*

Executing a spatial model in INLA requires the definition of a mesh on which the spatial term is defined, the resolution of which partly defines the precision of the results. The mesh for the Matérn correlation (SRF) terms were constructed with a maximum edge length (i.e., mesh resolution) of one-fifth of the estimated spatial range parameter, which defines the distance at which spatial dependency between nesting locations remains significant. This choice aimed to balance computational efficiency with the necessary resolution to capture spatial variability in the data. The estimated spatial range parameter of 7.5 km was the lowest value at which the *spatiotemporal* model could run, but lower (range = 4 km) and higher (range = 10 km) specifications for the *spatial* model did not result in substantial alterations to parameter estimates, spatial effects, or model fit. This specification resulted in mesh sizes for the spatial and spatiotemporal models of 6208 and 80704 (6208 x 13 knots), respectively. The model fitting process in INLA also requires specifying priors for the range (r) and variance (σ) of the spatial correlation. Following Simpson et al. (2017), we specified penalized complexity (PC) priors for r and σ, with the probability P(r < 7.5) = 0.05 and P(σ > 2) = 0.05. Default PC priors and hyperparameters according to the INLA manual (https://inla.r-inla-download.org/r-inla.org/doc/latent/) were used for the rw2 and iid splines, including U=1 for the rw2 splines (where “U” specifies the degree of smoothing) and σ = 1 for the iid splines, with the probability P(U > 1) = 0.05 and P(σ > 1) = 0.05, respectively.

*Fledgling Count Models*

For the *spatial* model of fledgling counts, the lowest possible spatial range parameter that would permit the model to run, of 4 km, was utilized based on the assumption that the dispersal abilities of pre-fledged chicks does not exceed this distance and that nesting sites are generally isolated by larger distances than this estimate. Accordingly, PC priors for r and σ were used for specifying the Matérn correlation term, with the probability P(r < 4) = 0.05 and P(σ > 2) = 0.05. The same priors for rw2 and iid splines as those used in the hatch success models (described above) were applied here.

Simpson D, Rue H, Riebler A, Martins T & Sørbye SH. 2017. Penalising model component complexity: A principled, practical approach to constructing priors. *Statistical Science* 32(1), 1-28. https://doi.org/10.1214/16-STS576
