# Supplementary figures and images for: Spatiotemporal and weather effects on the reproductive success of piping plovers on Prince Edward Island, Canada
Source: Ecol Evol. 2024 Aug 7;14(8):e11581. doi: 10.1002/ece3.11581 (PMC11303845; doi:10.1002/ece3.11581)

2011

2012

2013

2014

2015

2016

2017

2018

2019

2020

2021

2022

2023

Spatial smoother

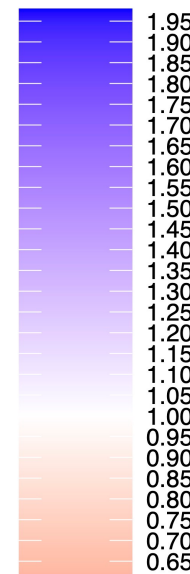

Supplement: Supplementary file 1 — Appendix S1. [file ECE3-14-e11581-s001.zip › Fig S1 - SD of SRF (revised).pdf]
